# Roles of membrane trafficking in plant cell wall dynamics

**DOI:** 10.3389/fpls.2015.00878

**Published:** 2015-10-19

**Authors:** Kazuo Ebine, Takashi Ueda

**Affiliations:** ^1^Department of Biological Sciences, Graduate School of Science, The University of Tokyo, Tokyo, Japan; ^2^Japan Science and Technology Agency, Precursory Research for Embryonic Science and Technology, Kawaguchi, Japan

**Keywords:** membrane trafficking, cell wall, transport mechanisms, RAB, exocyst, SNARE

## Abstract

The cell wall is one of the characteristic components of plant cells. The cell wall composition differs among cell types and is modified in response to various environmental conditions. To properly generate and modify the cell wall, many proteins are transported to the plasma membrane or extracellular space through membrane trafficking, which is one of the key protein transport mechanisms in eukaryotic cells. Given the diverse composition and functions of the cell wall in plants, the transport of the cell wall components and proteins that are involved in cell wall-related events could be specialized for each cell type, i.e., the machinery for cell wall biogenesis, modification, and maintenance could be transported via different trafficking pathways. In this review, we summarize the recent progress in the current understanding of the roles and mechanisms of membrane trafficking in plant cells and focus on the biogenesis and regulation of the cell wall.

## Introduction

Membrane trafficking is a key mechanism for transporting proteins, lipids, and polysaccharides among organelles in plant cells (Fujimoto and Ueda, 2012). The key molecules involved in membrane trafficking are generally conserved among eukaryotic cells, and the specific diversification of machinery components for membrane trafficking has occurred in plants and is associated with the development of plant-specific transport pathways during plant evolution (Fujimoto and Ueda, 2012). The cell wall is a characteristic structure of plant cells, and the components of this structure differ among organs and cell types in plants. Recent studies indicate that the diversification of membrane trafficking contributes to cell wall differentiation in plant cells (Kim and Brandizzi, 2014). Herein, we present an overview of the recent findings on the pivotal roles of membrane trafficking in the biogenesis and regulation of the cell wall.

## Molecular Mechanisms Underlying Membrane Trafficking in Plant Cells

**FIGURE 1:**
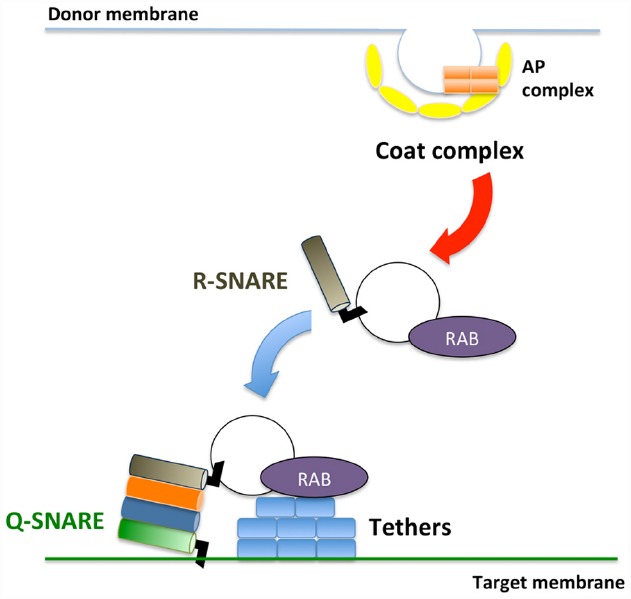
**Schematic framework of membrane trafficking.** Transport vesicles bud from donor membranes mediated through coat protein complexes. The formed vesicles are transported and subsequently tethered to the target membrane through RAB GTPases and tethers. At the final step, SNARE proteins execute membrane fusion between transport vesicles and target membranes.

Membrane trafficking is accomplished via three sequential steps: (1) vesicle budding from donor organelles, frequently involving coat protein complexes, such as COPI and COPII; (2) tethering the vesicles to the target membrane through activated Rab and Rab effectors; and (3) vesicle fusion with the target organelle, mediated through SNARE molecules (Ebine and Ueda, 2009; Fujimoto and Ueda, 2012; Figure 1). A majority of the proteins synthesized at the endoplasmic reticulum (ER) and polysaccharides synthesized in the Golgi are transported to the *trans*-Golgi network (TGN) and subsequently delivered to each organelle or the plasma membrane/extracellular space or traffic through the Golgi-independent trafficking pathway to the vacuole in plant cells (Pedrazzini et al., 2013; Inada and Ueda, 2014). The direct interaction between the ER and plasma membrane has also been reported (Staehelin, 1997; Sparkes et al., 2011; Wang et al., 2014; Perez Sancho et al., 2015), although the exchange of molecules at this contact point has not been demonstrated in plants.

Two Rab GTPases, RAB8/RABE and RAB11/RABA, have been implicated in the regulation of the pathway that mediates transport to the plasma membrane in plant cells (Speth et al., 2009; Fujimoto and Ueda, 2012; Inada and Ueda, 2014). In mammalian and yeast cells, these two Rab GTPases sequentially regulate transport to the plasma membrane, and the exocyst complex acts as a tethering complex in the RAB11–RAB8 cascade (Mizuno-Yamasaki et al., 2012). The exocyst complex comprises eight proteins that are responsible for tethering secretory vesicles to the plasma membrane (Synek et al., 2014). After the membrane vesicle is tethered to the plasma membrane, an R-SNARE protein on the vesicle and three Q-SNARE molecules on the plasma membrane assemble into a SNARE complex that executes membrane fusion between the membranes. Intriguingly, the number of genes encoding RAB11/RABA and a component of the exocyst complex, EXO70, is remarkably high in plants: 26 RAB11, classified into six subgroups (RABA1–RABA6), and 23 EXO70, classified into eight subgroups (EXO70A–EXO70H), are encoded in the *Arabidopsis* genome (Chong et al., 2010; Fujimoto and Ueda, 2012).

A functional analysis of the RAB11 proteins in *Nicotiana benthamiana* leaf epidermal cells indicated that RABA1b regulates the transport pathway from the TGN to the plasma membrane but that RABA4c regulates transport from the plasma membrane to the TGN (Choi et al., 2013). This result suggests that each Rab11 subgroup regulates different transport pathways. ARA6/RABF1 is also involved in transport to the plasma membrane, although the specific cargo remains unclear (Ebine et al., 2011). Each EXO70 subgroup has also been implicated in the regulation of a different transport pathway in plant cells (Synek et al., 2014). Exo70A1 regulates transport to the plasma membrane, whereas EXO70B1 regulates the transport of autophagosomes to vacuoles (Synek et al., 2006; Kulich et al., 2010, 2013). EXO70E2 regulates an unconventional secretory pathway mediated through an autophagosome-like double membrane structure termed EXPO (Wang et al., 2010; Ding et al., 2014). These EXO70 members likely share the same exocyst core complex and have been implicated as targets for ubiquitin-mediated degradation (Zarsky et al., 2013). Moreover, some RAB11 and EXO70 members are expressed in specific organs and are likely associated with the cell type-specific differentiation of membrane trafficking pathways (Lycett, 2008; Chong et al., 2010; Asaoka et al., 2012; Synek et al., 2014).

The SYP1 group contains members of the Q-SNARE family, which function in membrane fusion events at the plasma membrane and cell plate. SYP1 proteins have been classified into three subgroups in seed plants: SYP11, SYP12, and SYP13 (Saito and Ueda, 2009; Kanazawa et al., 2015). SYP111, which is also known as KNOLLE in *Arabidopsis*, localizes to the cell plate and mediates membrane fusion during cell division (Lauber et al., 1997; Muller et al., 2003). Both SYP12 and SYP13 localize to the plasma membrane. However, SYP12, but not SYP13, accumulates at the focal site of the cell, which reflects a functional difference between SYP12 and SYP13. Whereas SYP132, which is the most abundantly and ubiquitously expressed SYP13 protein in *Arabidopsis*, localizes uniformly at the plasma membrane of the growing pollen tube and root hair, SYP12 proteins localize to distinctive parts of the pollen tube and root hair. SYP123 localizes to the tip of the root hair, and SYP124 and SYP125 localize to distinct parts of the pollen tube (Enami et al., 2009; Silva et al., 2010; Ul-Rehman et al., 2011; Ichikawa et al., 2014). An interactomic analysis also revealed that SYP1 proteins interact with distinct sets of proteins (Fujiwara et al., 2014), further supporting the functional differentiation among SYP1 members.

In addition to proteinaceous transport machinery components, lipids play critical roles in membrane trafficking. Phosphatidylinositol derivatives (PIs) are key molecules that determine the characteristics of membrane domains (Fujimoto and Tsutsumi, 2014; Krishnamoorthy et al., 2014). Interactions between PIs and binding proteins depend on the phosphorylation state of PIs, and the phosphorylation status of PIs is tightly regulated through phosphatases and kinases (Fujimoto and Tsutsumi, 2014; Krishnamoorthy et al., 2014). Phosphatidylinositol 4-kinase (PI4K) and 5-kinase (PI5K) accumulate at the tips of tip-growing cells, where these enzymes induce the accumulation of PI(4,5)P2 and thereby contribute to the elongation of these cells and deposition of the cell wall materials at the tips by regulating actin dynamics (Preuss et al., 2006; Krishnamoorthy et al., 2014). These kinases also occasionally act as effector molecules of Rab GTPases; PI4Kβ is an effector molecule of RABA4, and PI5K2 is an effector molecule of RABE1d (Camacho et al., 2009; Szumlanski and Nielsen, 2009; Antignani et al., 2015), which indicates a tight link between Rab GTPase and PI metabolism.

Some proteins at the plasma membrane are endocytosed into the cytoplasm in response to the signals associated with extracellular conditions (Fujimoto and Tsutsumi, 2014). Clathrin-mediated endocytosis involves clathrin and dynamin-related proteins, where adapter protein complex 2 (AP-2) mediates cargo loading and clathrin assembly in eukaryotic cells (McMahon and Boucrot, 2011). Recent studies have shown that plants also use another adaptor complex called TPLATE (Van Damme et al., 2011; Gadeyne et al., 2014; Zhang et al., 2015). During NaCl stress, the clathrin-independent endocytosis of PIP2;1, an aquaporin residing on the plasma membrane, has also been reported in plants (Li et al., 2011; Chevalier and Chaumont, 2014).

## Membrane Traffic Regulating Cell Wall Deposition

**FIGURE 2:**
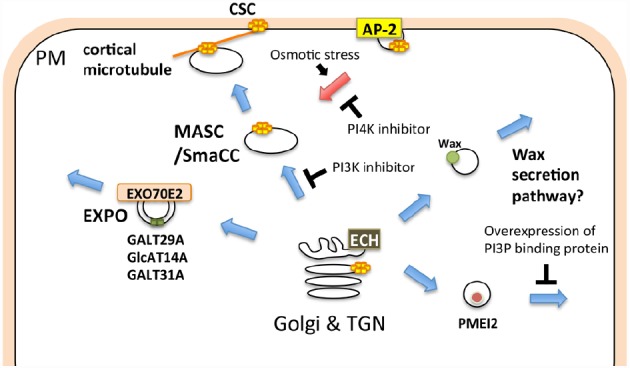
**Schematic illustration of membrane trafficking pathways involved in cell wall-related functions.** The cellulose synthase complex (CSC) is transported to the plasma membrane via the MASC (microtubule-associated cellulose synthase compartment)/SmaCC (small CESA-containing compartments), and is internalized through the AP-2-dependent endocytic pathway. The EXPO-mediated secretory pathway, ECH-dependent wax secretion, and PI3P-dependent secretion of PMEI2 also contribute to the biogenesis and/or regulation of the cell wall. Blue and red arrows indicate exocytic and endocytic pathways, respectively.

Many cell wall-associated molecules, including cellulose synthase (CESA), callose synthase (CALS)/glucan synthase like (GSL), and pectin methylesterase (PME), are transported to the plasma membrane and/or extracellular compartment via membrane trafficking. The cellulose synthase complex (CSC), comprising CESA, synthesizes cellulose at the plasma membrane. Cellulose deposition is strictly regulated depending on cell types, developmental stages, and environmental changes, thus the subcellular localization of the CSC, which profoundly depends on membrane trafficking, should be tightly regulated (McFarlane et al., 2014a). The CSC harbors a rosette-like structure, which has previously been detected in the ER of the moss, *Funaria hygrometrica* (Rudolph, 1987). This evidence might indicate that the rosette-shaped CSC is assembled at the ER and subsequently transported to the plasma membrane via the unique compartment, MASC (microtubule-associated cellulose synthase compartment)/SmaCC (small CESA-containing compartments; Sampathkumar et al., 2013; Gonneau et al., 2014; Hill et al., 2014; Kim and Brandizzi, 2014; McFarlane et al., 2014a; Figure 2). The endocytic transport of the CSC has also been observed in other events. For example, CSC internalization, which is mediated through AP-2, occurs in response to osmotic changes (Crowell et al., 2009; Bashline et al., 2013; Fujimoto et al., 2015; Figure 2). A recent study showed that CESA3 endocytosis is inhibited through phenylarsine oxide (PAO), an inhibitor of PI4K, whereas LY294002, an inhibitor of PI3K, blocks the transport of CESA3 from the Golgi apparatus (Fujimoto et al., 2015), indicating distinct requirements of PIs in distinctive trafficking steps in CSC transport (Figure 2).

CALS/GSL synthesizes callose at the plasma membrane or cell plate (Ferguson et al., 1998). Polysaccharides other than cellulose and callose are synthesized at the Golgi and subsequently transported to the extracellular space. The processing and/or modification of polysaccharides also occur during transport to the extracellular space (Schoberer and Strasser, 2011; Oikawa et al., 2013). ECHIDNA (ECH), which is required for TGN function, is also important for the secretion of polysaccharides (Gendre et al., 2011, 2013; McFarlane et al., 2013, 2014b). Both *ech* and *trs120-4*, a mutant of the predicted component of the RAB11 activation complex, exhibit a defect in general secretion, but only *ech* is defective in wax secretion (McFarlane et al., 2014b). Wax is synthesized at the ER and subsequently transported to the extracellular compartment (Haslam and Kunst, 2013). Thus, wax could be secreted via a different pathway than polysaccharides in plant cells (Figure 2). Furthermore, AtGALT31A, an arabinogalactan glycosyltransferase, localizes to Exo70E2-positive compartments, suggesting that this protein is secreted into the extracellular space through the EXPO (Poulsen et al., 2014; Figure 2). These results suggest that cell wall-related components are transported through diverse trafficking pathways. The involvement of the exocyst complex in secondary cell wall formation has also been reported. Exo70A1 is required for SCW formation during tracheary element formation (Li et al., 2013), and a recent study has also revealed a specific trafficking mechanism involving Exo70A1 in tracheary element formation (Oda et al., 2014).

The delivery of cell wall components is also strictly regulated during tip growth and cell plate formation. The CSC and CALS accumulate at the tip of the pollen tube (Cai et al., 2011), and this localization is maintained through targeted membrane trafficking at the tip of the pollen tube (Mollet et al., 2013; Onelli and Moscatelli, 2013). Pectin methylesterase inhibitor 2 (PMEI2) accumulates at the tip of the pollen tube, whereas pollen-specific pectin methylesterase1 (PPME1) localizes throughout the cell wall (Rockel et al., 2008). PMEI2 aggregates in the cytoplasmic compartment through the overexpression of the FYVE domain, a PI3P protein-binding domain. In contrast, the overexpression of the FYVE domain does not affect the secretion of PPME1 (Figure 2). These results indicate that PMEI2 and PPME1 are secreted through different pathways. In growing pollen tubes or root hair cells, many vesicles accumulate at the tip of the elongation zone, and these vesicles are labeled with RABA1 and/or RABA4 (Cole et al., 2005; de Graaf et al., 2005; Wen et al., 2005; Cole and Fowler, 2006; Asaoka et al., 2012; Caballero-Lima et al., 2013; Gu and Nielsen, 2013; Zhang et al., 2013), which suggests that these vesicles mediate secretion to the tips of growing cells, contributing to the focal elongation of these cells. RAB11, an ortholog of *Arabidopsis* RABA, regulates trafficking from the recycling endosome to the plasma membrane in animal cells (Wandinger-Ness and Zerial, 2014). Thus, either (or both) of the two subgroups of plant RAB11, RABA1, and RABA4 might regulate dynamic endocytosis and/or recycling at the tip region of the growing pollen tube.

The direction of membrane trafficking dynamically changes during cell plate formation (McMichael and Bednarek, 2013; Jurgens et al., 2015), and both secretory and endocytic pathways contribute to this process (McMichael and Bednarek, 2013; Jurgens et al., 2015). The adaptor complex 1 (AP-1) regulates transport to the vacuole in leaf cells and the transport of KNOLLE/SYP111 to the cell plate (Fujimoto and Ueda, 2012; Teh et al., 2013). A mutation in EXO84b, a component of the exocyst complex, induces the mislocalization of the polysaccharides recognized by JIM7 or LM14 in dividing cells in immunocytochemistry experiments (Rybak et al., 2014). This result indicates that exocyst-mediated secretion contributes to the transport of polysaccharides to the cell plate. The CSC and CALS also accumulate in the cell plate (Hong et al., 2001; Thiele et al., 2009; Miart et al., 2014). The CSC is transported to the newly formed cell wall via multiple pathways (Miart et al., 2014). Callose synthesis occurs at the cell plate during late anaphase, which is required for the completion of cell division (Thiele et al., 2009). CALS/GSL also accumulates at the cell plate (Hong et al., 2001), but the molecular mechanism of this localization remains unclear.

## Regulation of Membrane Composition and Trafficking via the Cell Wall

Organelle membranes in both animal and plant cells comprise many microdomains (Martiniere and Runions, 2013; Yoshida et al., 2013; Jarsch et al., 2014; Konrad et al., 2014). Recent studies suggest that the cell wall possesses regulatory functions for the structure and function of the plasma membrane. The mobility of microdomains in the plasma membrane is regulated through interactions with actin filaments, and actin filament dynamics are regulated through PI(4,5)P2 in animal cells (Kusumi et al., 2005; Krishnamoorthy et al., 2014). In plant cells, interactions with the cell wall likely represent key regulatory mechanisms for some microdomains (Martiniere et al., 2012; Martiniere and Runions, 2013). PIN1, an auxin efflux carrier, localizes to microdomains in the plasma membrane, and mutations in CESA3 induce the mislocalization of PIN1, expressed under the regulation of the PIN2 promoter (Feraru et al., 2011). A recent analysis of PMR4, a CALS that acts during the plant defense response, indicated that PMR4 interacts with RABA4c, promoting the localization of RABA4c at the plasma membrane (Ellinger et al., 2014). This result could represent a functional link between the focal accumulation of callose and targeted secretion upon pathogen attack. Thus, the integrity and/or composition of the cell wall and the membrane trafficking system affect each other, further indicating the tight linkage and mutual regulation between the plant cell wall and membrane trafficking.

## Perspective

As described above, various aspects of interaction between the plant cell wall and membrane trafficking have been reported. However, the underlying mechanisms remain unclear. In particular, the feedback mechanism of the regulation of membrane trafficking via the cell wall has essentially remained unexplored. There are also many open questions concerning the molecular mechanisms underlying the transport of cell wall components through distinct trafficking pathways. For example, how and where are cell wall components sorted for loading onto different trafficking intermediates? Further extensive research in this area is needed to obtain a precise understanding of the tight linkage between the cell wall and membrane trafficking.

### Conflict of Interest Statement

The authors declare that the research was conducted in the absence of any commercial or financial relationships that could be construed as a potential conflict of interest. The Review Editor Samantha Vernhettes declares that, despite having collaborated with the author Takashi Ueda within the past two years, the review was handled objectively. The Guest Associate Editor Masaru Fujimoto declares that, despite being affiliated with the same institute as the author Kazuo Ebine, the review process was handled objectively.
